# Hip adduction and abduction strength profiles in elite and sub-elite female soccer players according to players level and leg limb-dominance

**DOI:** 10.1186/s13102-024-00838-0

**Published:** 2024-02-22

**Authors:** Eloy Jaenada-Carrilero, Juan Vicente-Mampel, Luis Baraja-Vegas, Kristian Thorborg, Eloína Valero-Merlos, Paula Blanco-Gímenez, Pedro Gargallo, Iker J. Bautista

**Affiliations:** 1 Doctoral School, Catholic University of Valencia Saint Vincent Martyr, Valencia, Spain; 2 Faculty of Science Health, Catholic University of Valencia, Physiotherapy Department., C/ Ramiro de Maetzu 14, Torrent Valencia, Spain; 3 Department of Sports, Department of Orthopedic Surgery, Orthopedic Research Center-Copenhagen (SORC-C), Amager-Hvidovre Hospital, Copenhagen University Hospital, Copenhagen, Denmark; 4 Faculty of Nursing. Campus Jerónimos, Catholic University of Murcia, Murcia, Spain; 5 Institute of Sport, Nursing and Allied Health, University of Chichester, Chichester, UK

**Keywords:** Adduction:abduction ratio, Isometric contractions, Groin, Women’s football

## Abstract

**Background:**

Understanding the hip adduction and abduction strength in female soccer players is crucial for performance enhancement and injury prevention. This study compares the strength profiles in these muscle groups between elite and sub-elite female soccer players and assesses the impact of leg limb-dominance.

**Methods:**

A descriptive-comparative study was employed. Eighty-two female soccer players were evaluated. Isometric hip-adduction and abduction strength were measured using a handheld dynamometer.

**Results:**

Female elite and sub-elite soccer players displayed a mean and standard deviation (SD) on isometric hip-adductor strength for dominant (3.19 Nm/kg ± 0.69 vs. 2.40 Nm/kg ± 0.67) and non-dominant leg (3.32 Nm/kg ± 0.76 versus 2.42 Nm/kg ± 0.70), respectively. For isometric hip-abductor strength in elite and sub-elite players, a mean and SD of dominant (2.86 Nm/kg ± 0.56 vs. 2.07 Nm/kg ± 0.50) and non-dominant (2.80 Nm/kg ± 0.59 vs. 2.04 Nm/kg ± 0.43). In essence, elite players were stronger than sub-elite players on isometric hip-adduction (mean difference [MD] = 0.82 Nm/kg, CI_95%_ = 0.42–1.12) and abduction (MD = 0.83 Nm/kg, CI_95%_ = 0.54- 1.12) both in dominant and non-dominant, leg, whereas no differences existed for hip adduction:abduction ratios between groups and legs.

**Conclusions:**

Elite female athletes exhibited greater strength than sub-elite female players in both hip adduction and abduction, whereas adduction:abduction ratio values did not differ between the two groups or between different legs.

**Supplementary Information:**

The online version contains supplementary material available at 10.1186/s13102-024-00838-0.

## Background

Characterizing the physical condition profiles of athletes of various sports and performance levels, especially in terms of their strength profiles, is essential. The hip complex has been comprehensively studied in male soccer players [1-3]. The development of the musculature involved around the hip complex (i.e., adductor and abductor muscles) are essential for performance (e.g., sprinting and change of direction) [4]. and injury prevention (e.g., knee/groin injuries) [5]. Indeed, hip and groin injuries are frequent in professional men’s soccer players [6]. While several previous studies have suggested that groin pain is occurs less frequently in women’s soccer compared to men’s, it still represents a major issue in the women’s game as well [7, 8]

One of the most valuable parameters to characterize the strength profile in athletes of predominantly lower-limb sports, such as soccer, is the isometric hip strength assessment, which is an important part of the clinical examination of the hip and groin area [9, 10]. Previous studies have reported that specific isometric and eccentric hip strength tests can identify players who have sustained groin injuries [11, 12]. It has been suggested that long-lever isometric hip adduction squeeze strength, along with a history of acute groin injury, plays an important role in predicting the likelihood of a new groin injury [13]. In Spanish male footballers, Esteve et al. [14]. found that there were no overall differences between those with and without past-season groin pain for hip adductor squeeze strength. However, those athletes with past-season groin pain showed reduced scores on the hip adductor squeeze strength test, compared with those without past-season groin pain. These results were corroborated by Esteve et al. [15] which found that players who had experienced groin pain in the previous season were 2.4 times more likely to encounter a groin problem. Nonetheless, these authors found that the risk could reduce by 35% per unit (Nm/kg) increase in the long-lever adductor strength.

In professional ice hockey athletes, Tyler et al. [12]. showed that those players whose adductors were markedly weaker than their abductors were more likely to experience an adductor strain. This underscores the potential importance for the adduction:abduction ratio as a variable of consideration. In this sense, in male soccer players, the hip dominant side has been shown to be marginally stronger than the contralateral side, both in isometric hip adduction and abduction [1]. However, some larger studies have not been able to confirm that, showing no difference between dominant and non-dominant leg in male soccer players. For instance, Mosler et al. [16]. assessed a large cohort of asymptomatic male professional soccer players (*n* = 394) showing no differences in leg dominance, history of injury and ethnicity. At this point, in female soccer players, there exists a paucity of research data that associates risk factors with developing groin injuries. Specifically in female elite players from Australian Football, Mentiplay et al. [17]. reported no clinically relevant effect of limb dominance after evaluating more than 80 elite female players. Nevertheless, although previous studies in male soccer players have shown that hip strength ratios vary between dominant vs. non-dominant leg, little is known about the importance of player performance level, dominance, and adduction:abduction ratio in a cohort of female’s soccer players.

Currently, there are no available studies that specifically compare isometric hip adduction/abduction ratios in female soccer players across the players performance level (i.e., elite vs. sub-elite) and leg-dominance (i.e., preferred leg vs. non-preferred leg). Therefore, in this study we aimed (1) to describe and compare the isometric adduction/abduction strength profiles in elite and sub-elite female soccer players. We hypothesized that will be significant differences in isometric adduction and abduction strength profiles across different playing levels, based on the study of Prendergast et al. [18]. The second purpose was (2) to analyze the influence of player performance level (i.e., elite vs. sub-elite) and leg dominance (i.e., dominant vs. non-dominant leg) on hip adduction and abduction strength, as well as adduction:abduction ratio in female soccer players. Isometric adduction/abduction strength profiles according to lower limb dominance in female Australian footballers was studied by Mentiplay et al. [17]. showing no differences. Since both sport (i.e., Australian football and football) are similar in terms of physical demands, we hypothesized that there would be no significant differences in hip strength profiles according to leg dominance.

## Methods

### Study Design

A descriptive-comparative study was conducted to examine the isometric hip strength profile (i.e., adduction and abduction) and adduction:abduction strength ratio. This comparison was made across players performance level (i.e., elite vs. sub-elite soccer players) and lower-limb dominance (i.e., dominant vs. non-dominant leg).

### Participants

A total of 84 female soccer players from a professional soccer club in Spain were potentially eligible from 2019 until 2022. Finally, 82 players participated part in this study. A total of two players (*n* = 2) were unable to complete the test due to experiencing pain during the administration of the tests. All players were recruited from the same soccer club. Starting from the first year of evaluation (i.e., 2019), only new players who had not been previously tested were included in the study. Participants characteristics measured are presented in Table 1. Before testing procedures, all players provided a written informed consent. Players who had sustained lower limb injuries lasting more than 4 weeks in the last 3 months were excluded. Additionally, players with a history of athletic pubalgia within the last year were specifically excluded from the study. The classification of the group’s performance was based on the participant classification framework developed by McKay et al. [19]. That is, elite players, who compete at the international level (1st division and international championships), were distinguished from sub-elite players (i.e., highly trained/National level).

**Table 1:** Descriptive statistics (mean ± SD) of anthropometrical characteristics of the female players included in the study according to players’ level

	**Elite Female soccer players**	**Sub-elite Female soccer players**	***p*-value**
**Participants (n)**	44	38	nc
**Age (years)**	22.52 (4.79)	18.53 (2.72)	< 0.001
**Height (cm)**	166.79 (4.51)	165.84 (4.86)	0.360
**Body mass (kg)**	59.86 (5.75)	60.37 (6.20)	0.703
**Hours per week training**	7.5	6	nc
**Dominance (R/L)**	37/7	35/3	nc

*R* = right leg; *L* = left leg, *nc* = not calculated

### Procedures

Players underwent testing between July and August across three consecutive pre-season training periods (2019/20, 2020/21, 2021/22). All assessments of hip strength were conducted by the same physiotherapist to minimize potential sources of error. A portable hand-held dynamometer (HHD) (MicroFET 2, Hoggan Scientific, LLC, Salt Lake City, UT) was utilized for the hip strength assessments, with calibration performed before testing. This calibration process included zeroing the device. Furthermore, in each season, the HHD underwent additional calibration by setting it up with a known load to ensure consistent and accurate measurements over time. Maximal voluntary isometric hip adduction and hip abduction force, in both dominant and non-dominant legs were tested. All assessments were done on a massage table. The order in which the tests were conducted was varied systematically among participants. By doing so, we aimed to eliminate or reduce potential biases due to the order of tests and any transference effects that might occur if one test influenced the performance in subsequent tests. Specifically, each player completed a sequence of tests in the order of A, B, B, A, where 'A' represents abductor test and 'B' denotes adductor test. Subsequently, the average of the two 'A' conditions was calculated, and the same process was applied for the 'B' conditions. Two sub-maximal familiarization trials were performed to ensure the players were performing the correct action of pushing into the belt and the HHD. Verbal encouragement was provided during the test execution with a standard instruction of “*push, push, push*”. Prior to each testing period, players performed a standardized warm-up, which consisted of 5 min of stationary bike after 10 repetitions of concentric and eccentric abductor and adductor movements. After a short break of 5 min, players were tested.

Isometric hip adduction and hip abduction were measured in the supine position as introduced by Thorborg et al [9]. The participants were placed in the supine position and were told to stabilize themselves by holding onto the side of the table with their hands. For the adductor measurement, the examiner (E.J.) applied resistance in a fixed position, 2 cm proximal to the edge of the medial malleolus. The abductor measurement was performed using the HHD and a belt-fixation proximal to the edge of the lateral malleolus [20]. The participant being tested exerted a 5 s maximum isometric voluntary contraction against the HHD.

Two trials in the same leg were completed with a 30-s rest period between repetitions. The mean of peak force (measured in Newtons [N]) recorded for each limb across two attempts was used for data analysis. If variability between trials were more than 10%, a new trial was done. A reliability study performed in 10 female soccer players, selected randomly from our sample, showed that the intrarater reliability was found to be excellent according to the index correlation coefficient model 2.k (ICC_2.k_) = 0.86 (0.76 to 0.95) and standard error of measurement (SEM) of 0.26 Nm/kg for hip adduction and excellent, ICC_2.k_ = 0.80 (0.56 to 0.91) and SEM = 0.41 Nm/kg, for hip abduction. In addition, a recent reliability study showed excellent reliability coefficients (ICC = 0.92 to 0.96) and nearly perfect validity scores (*r* = 0.996) in comparison to fixed-frame dynamometry system [21].

### Statistical analysis

All results were expressed as a mean and standard deviation (± SD). Normality and homogeneity of variance assumptions were analyzed using Shapiro–Wilk test and Levene test, respectively. Relative reliability was examined using ICC_2.k_, whereas absolute reliability was calculated using SEM [22]. There was a statistically significant association (*p* < 0.001) between age of participants and their relative hip isometric adduction and abduction strength test scores (with correlation coefficients ranging from 0.30 to 0.45, all of which were statistically significant). As a result, the age factor was included as a covariate. Consequently, to examine the effect of players performance level (i.e., elite vs. sub-elite) and dominance (i.e., dominant vs. non-dominant leg) an analysis of covariance variance (ANCOVA, 2 × 2) was employed. For adduction:abduction ratio, an ANOVA (2 × 2) was performed as the Pearson correlation coefficient between adduction:abduction ratio and age were not statistically significant. Post hoc tests, utilizing the Bonferroni correction, were conducted to address multiple comparison. All post hoc analysis was presented using mean differences (MD) and 95% of confident intervals (CI_95%_). Effect size (ES) was calculated according to Cohen formulas [23]. and considered trivial (< 0.20), small (0.20 – 0.59), moderate (0.60 – 1.19), large (1.20 – 1.99), and very large (> 2.00) [24]. The statistically significant level was set at *p* < 0.05. All calculations were done using a statistical analysis tool (JASP v.0.17.1, the Netherlands).

## Results

**Fig. 1:**
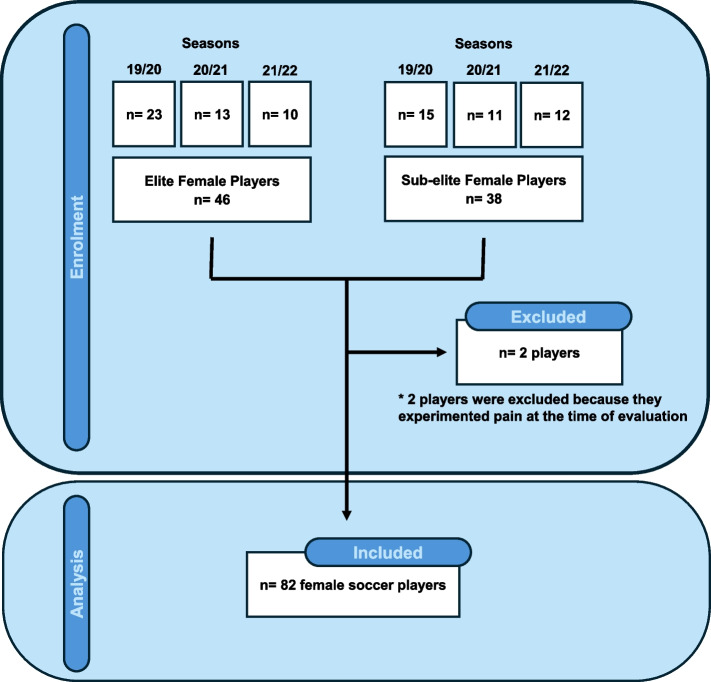
Flow diagram with the number of participants included in the study

Of the eighty-four players with potential to be evaluated in this study, finally eighty-two players were recruited. Two players were excluded due to be involved in the rehabilitation process of a previous injury. Therefore, a total of forty-six (*n* = 46) elite female players were evaluated during seasons 19/20 (*n* = 23), 20/21 (*n* = 13) and 21/22 (*n* = 10), meanwhile thirty-eight (*n* = 38) sub-elite female players were evaluated during seasons 19/20 (*n* = 15), 20/21 (*n* = 11) and 21/22 (*n* = 12) (Fig. 1).

Finally, eighty-two female soccer players completed the isometric hip adduction and abduction strength test. From final sample size, *n* = 44 (53%) were categorized as elite soccer players and *n* = 38 (47%) were categorized as sub-elite soccer players according to McKay, Stellingwerff, Smith, Martin, Mujika, Goosey-Tolfrey, Sheppard and Burke [19] classification (see Table 1).

Descriptive statistics (i.e., mean, SD, min, max and percentiles) of isometric hip strength and adduction:abduction ratio is described in Table 2. In addition, absolute values of isometric hip strength are summarized in Supplementary File 1.

**Table 2:** Descriptive statistics (mean, SD, minimum and maximum and percentiles) of hip isometric strength test for adductor, abductors, and adduction:abductor ratio in elite and sub-elite female soccer players

	**Elite female soccer players**			**Sub- Elite female soccer players**		
	**Hip adductor (Nm/kg)**	**Hip abductor** **(Nm/kg)**	**Ratio**	**Hip adductor** **(Nm/kg)**	**Hip abductor** **(Nm/kg)**	**Ratio**
**Dominant leg**						
Mean ± SD	3.19 ± 0.69	2.86 ± 0.56	1.13 ± 0.23	2.40 ± 0.67	2.07 ± 0.50	1.21 ± 0.43
Min – max	1.73 – 4.51	1.71 – 3.80	0.73 – 1.87	0.87 – 3.81	0.91 – 2.97	0.70 – 2.93
25th	2.77	2.41	0.97	2.07	1.75	0.92
50th	3.15	2.91	1.11	2.34	1.99	1.13
75th	3.73	3.35	1.24	2.82	2.47	1.41
**Non-dominant leg**						
Mean ± SD	3.32 ± 0.76	2.80 ± 0.59	1.20 ± 0.22	2.42 ± 0.70	2.04 ± 0.43	1.21 ± 0.43
Min – max	1.84 – 4.89	1.53 – 4.22	0.81 – 1.87	0.73 – 3.56	1.04 – 2.77	0.64 – 3.11
25th	2.74	2.47	1.06	2.04	1.84	1.00
50th	3.23	2.78	1.18	2.49	2.06	1.17
75th	4.07	3.21	1.28	2.84	2.29	1.39

Ratio was calculated as adduction relative strength divided by abduction relative strength

### Isometric Hip adduction test

**Fig. 2:**
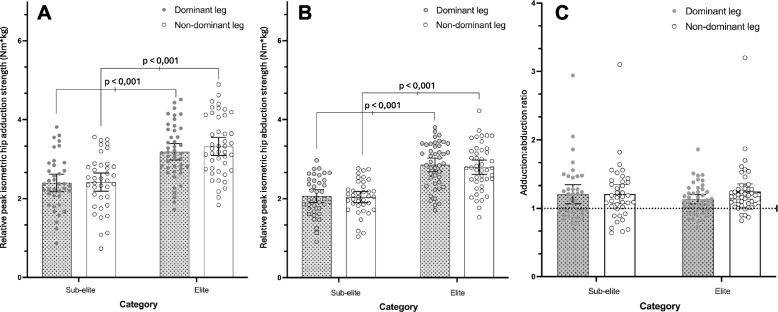
Relative peak isometric hip adduction (**A**) and abduction (**B**) and adduction:abduction ratio (**C**) results for the dominant and non-dominant leg for elite and sub-elite female soccer players. Dotted line in figure C represents an abduction:adduction ratio of 1.00. The whiskers represent 95% of confident interval

After adjusting for players´ age, the ANCOVA for isometric hip adduction revealed that there were significant differences (*p* < 0.001) for players performance level. On the other hand, no significant differences were found in the interaction between dominance x players performance level (*p* = 0.121). Independently from leg dominance, Bonferroni post hoc comparison revealed that elite players were stronger than their sub-elite counterparts (*p* < 0.001, MD = 0.89 Nm/kg, CI_95%_ = 0.60 to 1.18, ES = 0.67 [moderate]). Regarding leg dominance factor, no differences were found on preferred leg for elite female soccer players vs. non-preferred leg (*p* = 0.070, MD = -0.14 Nm/kg, CI_95%_ = -0.29 to 0.01). In sub-elite soccer players, there were no statistically significant differences regarding dominance (*p* = 1.000, MD = -0.02 Nm/kg, CI_95%_ = -0.17 to 0.15). Finally, elite female soccer players displayed stronger values in preferred leg (*p* < 0.001, MD = 0.82 Nm/kg, CI_95%_ = 0.42 to 1.12) and non-preferred leg (*p* < 0.001, MD = 0.95 Nm/kg, CI_95%_ = 0.54 to 1.36) in comparison with sub-elite female soccer players, see Fig. 2A.

### Isometric hip abduction test

The ANCOVA conducted for isometric hip abduction revealed significant differences for players level (*p* < 0.001) while no significant effects were observed for dominance main effect (*p* = 0.589) nor dominance x player`s performance level (*p* = 0.567). Bonferroni post hoc comparison revealed that elite players were stronger than their sub-elite counterparts (*p* < 0.001, MD = 0.82 Nm/kg, CI_95%_ = 0.61 to 1.02, ES = 0.87 [large]). Regarding dominance, the mean values of preferred leg for elite female soccer players were equal to the mean value in non-preferred leg (*p* = 0.872, MD = 0.06 Nm/kg, CI_95%_ = -0.05 to 0.16). In addition, in sub-elite soccer players there were no statistically significant differences regarding dominance factor (*p* = 1.000, MD = 0.02 Nm/kg, CI_95%_ = -0.09 to 0.14). Finally, elite female soccer players exhibited stronger values in preferred leg (*p* < 0.001, MD = 0.83 Nm/kg, CI_95%_ = 0.54 to 1.12) and non-preferred leg (*p* < 0.001, MD = 0.80 Nm/kg, CI_95%_ = 0.51 to 1.09) in comparison with sub-elite female soccer players see Fig. 2B.

### Isometric hip adduction:abduction ratios

No differences were observed in the isometric hip adduction:abduction ratio between players level and dominance (MD = -0.04 [-0.18 to 0.10], *p* = 0.558 and MD = -0.04 [-0.08 to 0.10], *p* = 0.121), respectively. The isometric hip adduction and abduction strength profiles for both preferred and non-preferred leg, of elite and sub-elite female soccer players are displayed in Fig. 2.

## Discussion

This study aimed to compare the isometric hip adduction/abduction strength profile of elite and sub-elite female soccer players, while also analyzing the influence of limb-dominance. The primary finding revealed that elite female soccer players exhibited stronger values than sub-elite counterparts in both the isometric hip adduction and abduction test. Furthermore, no significant differences were observed between dominant and non-dominant leg, as well as in the adduction:abduction strength ratios, in both elite and sub-elite players.

Modern soccer is characterize by numerous short-speed actions mixed with large number of acceleration/decelerations actions as well as sudden changes of directions [25, 26]. However, these physically demanding actions requires a well-developed neuromuscular system. In our study, elite female soccer players demonstrated strongest values for adductor and abductor isometric hip strength in comparison to sub-elite players, even when the results were adjusted by players´ age, as shown in Fig. 2. Similar findings were observed by Prendergast et al. [18]. in a study involving male Australian footballers. In that research, elite male athletes exhibited higher values of adduction and abduction strength test in comparison with sub-elite and amateur athletes. These differences in performance between players level (i.e., elite vs. sub-elite) could be attributed to the number of weekly training sessions and match/training intensity.

Concerning players` leg limb-dominance, our results indicated no influence of preferred leg on hip adduction or abduction strength test in elite vs. sub-elite female soccer players, as detailed in Table 2. For instance, in a sample of male elite soccer players, Thorborg et al. [1]. identified marginal differences in hip adduction and abduction strength regarding dominance. However, they concluded that the negligeable difference between the dominant and non-dominant side was within the measurement variation of the test procedure. Our findings, in the context of female players, suggest that there is symmetry in isometric hip adduction and abduction strength, regardless of the leg dominance. This symmetry could have important clinical implications, especially in the recovery process after an injury. Given this symmetry, the strength of the non-involved limb can be effectively used as a reliable benchmark in setting rehabilitation goals. This approach ensures that the recovery process is both tailored to the individual athlete and balanced, leading to more effective rehabilitation outcomes.

Specifically in female athletes, although in other sport, Mentiplay et al. [17]. revealed no clinically relevant effect of leg-limb dominance on isometric strength of hip adduction and abduction. Collectively, these results are consistent with our findings, which showed no effect of leg dominance on isometric strength profile of the hip. Our study revealed that, regardless of players level (i.e., elite vs. sub-elite), no differences were observed in the adduction:abduction ratios for both the preferred and non-preferred leg (see Fig. 2C). The mean values for the hip adduction:abduction ratio for elite and sub-elite female players ranged from 1.13 – 1.21 (see Table 2). These results indicate slightly higher ratios compared to those reported by Thorborg et al. [1]. in male soccer players (1.04 – 1.06) and by Prendergast et al. [18]. in Australian footballers (1.03 – 1.13). This suggest that a deficit in hip abduction strength could account for the observed differences in isometric hip strength ratios between sexes. However, our findings also indicated that while isometric hip strength varies between players performance level, the adduction:abduction ratios remained consistent, showing no significant differences based on players performance or preferred limb. In the context of female athletes, Mentiplay et al. [17]. reported a median of 1.00 in dominant and non-dominant leg in elite Australian footballers. Previous studies conducted in a cohort of male soccer players have demonstrated that reduced adduction strength has been associated with groin injuries [11]. Similarly, Roe et al. [3]. showed that there was an association between changes in adductor strength and distance covered at high-sprint after a competitive match. However, it should be note that little is known about the isometric hip adductor and abductor strength profiles and their relationship with groin injuries and/or players performance in a cohort of female soccer players. Additionally, the association with other types of injuries, such as ACL injuries, should be considered. Abductor strength is crucial for frontal plane stability and given the higher incidence of these injuries among female players [27]. this relationship warrants further exploration. Further studies, in elite female soccer players, need be addressing these questions.

Several limitations to this study should be keep in mind when interpreting the findings. Although the assessments were consistently performed by the same physiotherapist performed to ensure an intra-rater consistency, we did not use a fixed-frame dynamometry system to measure isometric hip adduction and abduction, which could have helped in eliminating any potential intra-tester biases. Previous studies have demonstrated that HHD is a valid tool to assess isometric hip strength [28]. even when compared to fixed-frame dynamometry system [21]. However, to enhance the quality to our data, we conducted a test–retest reliability showing an excellent reliability result both adduction and abduction isometric hip strength. Finally, the assessment period in this study corresponded to pre-season. Although all the players had a special routine during off-season period, the differences obtained in isometric hip strength could differ during the competition period.

All in all, this study reported that elite female soccer players were stronger than sub-elite female players in hip adduction and abduction strength test whereas adduction:abduction ratio values did not differ between players performance (i.e., elite vs. sub-elite) or legs (i.e., dominant vs. non-dominant).

## Conclusions

The isometric hip adduction/abduction strength profile of female soccer players was compared across different performance level as well as lower-limb dominance. While no significant differences were observed in limb dominance within category, large differences were found between elite vs. sub-elite isometric hip strength profiles. Additionally, although differences were found across players performance level on main variables (i.e., adduction and abduction isometric hip strength), no significant differences were found in adduction:abduction strength ratio. Furthermore, our findings suggest that female strength ratios were slightly higher in comparison to those reported in male soccer players. This could potentially account for the lower incidence of groin injury in female relative to their male counterparts.

### Supplementary Information

**Additional file 1:** **Supplementary File 1.** Descriptive values of isometric hip adduction and abduction strength test, **Table S1.** Descriptive statistics (mean, standard deviation, minimum and maximum and percentiles) of isometric hip strength test for adductor and abductors.

## Data Availability

Descriptive values of isometric hip adduction and abduction strength test are available in appendix A “Supplementary File 1”. The datasets used and/or analysed during the current study are available from the corresponding author on reasonable request.
