# Supplementary material for: Hip adduction and abduction strength profiles in elite and sub-elite female soccer players according to players level and leg limb-dominance
Source: BMC Sports Sci Med Rehabil. 2024 Feb 22;16:53. doi: 10.1186/s13102-024-00838-0 (PMC10882891; doi:10.1186/s13102-024-00838-0)
Supplement: Supplementary file 1 — Additional file 1: Supplementary File 1. Descriptive values of isometric hip adduction and abduction strength test, Table S1. Descriptive statistics (mean, standard deviation, minimum and maximum and percentiles) of isometric hip strength test for adductor and abductors. [file 13102_2024_838_MOESM1_ESM.docx]

**Supplementary File 1.** Descriptive values of isometric hip adduction and abduction strength test.

**Table S1.** Descriptive statistics (mean, standard deviation, minimum and maximum and percentiles) of isometric hip strength test for adductor and abductors.

|  | **Elite female soccer players** | | **Sub- Elite female soccer players** | |
| --- | --- | --- | --- | --- |
|  | **Hip adductor (N)** | **Hip abductor (N)** | **Hip adductor (N)** | **Hip abductor (N)** |
| **Dominant leg** |  |  |  |  |
| Mean (SD) | 165 (31.12) | 150 (26.82) | 122 (30.63) | 109 (23.08) |
| Min – max | 92 – 236 | 93 – 197 | 56 – 182 | 55 – 188 |
| 25^th^ | 141 | 95 | 99 | 95 |
| 50^th^ | 164 | 109 | 119 | 109 |
| 75^th^ | 184 | 121 | 151 | 121 |
| **Non-dominant leg** |  |  |  |  |
| Mean (SD) | 169 (31.74) | 149 (34.16) | 123 (32.42) | 109 (23.08) |
| Min – max | 108 – 240 | 88 – 251 | 47 – 176 | 55 – 176 |
| 25^th^ | 150 | 124 | 110 | 96 |
| 50^th^ | 174 | 149 | 119 | 107 |
| 75^th^ | 188 | 170 | 150 | 124 |
